# Matrix Metalloprotease 3 Activity Supports Hippocampal EPSP-to-Spike Plasticity Following Patterned Neuronal Activity via the Regulation of NMDAR Function and Calcium Flux

**DOI:** 10.1007/s12035-016-9970-7

**Published:** 2016-06-28

**Authors:** Patrycja Brzdąk, Jakub Włodarczyk, Jerzy W. Mozrzymas, Tomasz Wójtowicz

**Affiliations:** 1 Laboratory of Neuroscience, Department of Biophysics, Wroclaw Medical University, Chalubinskiego 3, Wroclaw, 50-368 Poland; 2 Department of Animal Molecular Physiology, Institute of Experimental Biology, Wroclaw University, Wroclaw, Poland; 3 Laboratory of Cell Biophysics, Department of Molecular and Cellular Neurobiology, Nencki Institute of Experimental Biology, Warsaw, Poland

**Keywords:** Matrix metalloprotease, Extracellular proteolysis, Synaptic plasticity, NMDAR, E-S potentiation, Hippocampus

## Abstract

Matrix metalloproteases (MMPs) comprise a family of endopeptidases that are involved in remodeling the extracellular matrix and play a critical role in learning and memory. At least 24 different MMP subtypes have been identified in the human brain, but less is known about the subtype-specific actions of MMP on neuronal plasticity. The long-term potentiation (LTP) of excitatory synaptic transmission and scaling of dendritic and somatic neuronal excitability are considered substrates of memory storage. We previously found that MMP-3 and MMP-2/9 may be differentially involved in shaping the induction and expression of excitatory postsynaptic potential (EPSP)-to-spike (E-S) potentiation in hippocampal brain slices. MMP-3 and MMP-2/9 proteolysis was previously shown to affect the integrity or mobility of synaptic *N*-methyl-d-aspartate receptors (NMDARs) in vitro. However, the functional outcome of such MMP-NMDAR interactions remains largely unknown. The present study investigated the role of these MMP subtypes in E-S plasticity and NMDAR function in mouse hippocampal acute brain slices. The temporal requirement for MMP-3/NMDAR activity in E-S potentiation within the CA1 field largely overlapped, and MMP-3 but not MMP-2/9 activity was crucial for the gain-of-function of NMDARs following LTP induction. Functional changes in E-S plasticity following MMP-3 inhibition largely correlated with the expression of cFos protein, a marker of activity-related gene transcription. Recombinant MMP-3 promoted a gain in NMDAR-mediated field potentials and somatodendritic Ca^2+^ waves. These results suggest that long-term hippocampal E-S potentiation requires transient MMP-3 activity that promotes NMDAR-mediated postsynaptic Ca^2+^ entry that is vital for the activation of downstream signaling cascades and gene transcription.

## Introduction

In the central nervous system, several forms of experience-dependent plasticity (i.e., substrates of learning and memory) require the activity-dependent control of synaptic efficacy. In a classic (although not unique) mechanism, *N*-methyl-d-aspartate receptors (NMDARs) gate Ca^2+^ influx following membrane depolarization during episodes of neuronal activity and determine the extent of synaptic long-term potentiation (LTP) or long-term depression (LTD) that subsequently develops [1]. α-Amino-3-hydroxy-5-methyl-4-isoxazolepropionic acid receptors (AMPARs) mediate the majority of synaptic currents at excitatory synapses. Most studies on the mechanisms of excitatory synapses have focused on changes in AMPAR-mediated signals. However, the NMDAR-mediated component may reveal various forms of plasticity and directly or indirectly influence neuroplastic changes at different levels of neuronal processing. For example, impairments in long-term associative memory that are induced by blocking NMDARs after behavioral training [2] clearly demonstrate that the role of these receptors goes well beyond their well-established function in LTP_AMPA_ induction. Moreover, the number and/or subunit composition of synaptic NMDARs may be regulated by neuronal activity and sensory experience [3], and the plasticity of the NMDAR component might subsequently influence the plasticity of the AMPAR component [4]. Additionally, in an intact acute brain slice preparation, the NMDAR component of field excitatory postsynaptic potentials (fEPSPs) in response to low-frequency stimulation was barely detectable, or other conductances (e.g., γ-aminobutyric acid [GABA]ergic transmission and outward potassium currents) may mask NMDAR-related synaptic currents [5, 6]. However, no systematic studies have evaluated the temporal effect of NMDARs on hippocampal plasticity. In addition to synaptic plasticity, memory storage may involve multiple levels of long-term modifications of neuronal input-output properties through far more complex mechanisms than LTP-LTD_AMPA_ alone. Neurons can significantly enhance information storage capacity by scaling dendritic and somatic excitability and learning [7, 8]. A hallmark of such a phenomenon occurs during tetanically evoked synaptic LTP when the probability of firing an action potential in postsynaptic neurons increases beyond the probability that is predicted by an increase in synaptic input (excitatory postsynaptic potential (EPSP)-to-spike potentiation; E-S plasticity [9, 10]). Although synaptic and nonsynaptic plasticity differs in the mechanism of expression, these processes share the common requirements of NMDAR activation and rise in postsynaptic Ca^2+^. However, the temporal requirement for NMDAR activity in synaptic LTP_AMPA_ and E-S potentiation remains unknown.

Enhancements in neuronal activity are associated with the release of specific factors that further support the maintenance of synaptic plasticity. The activity of extracellular matrix metalloproteases (MMP), a family of zinc endopeptidases, was shown to play a crucial role in learning and memory [11, 12]. To date, at least 24 MMP subtypes have been identified in the brain, including secreted and membrane-bound subtypes, and dozens of MMP substrates have been identified in vitro [11]. However, remaining largely unknown is the cellular mechanism of such MMP subtype-specific proteolysis in hippocampal plasticity. The best characterized MMPs in neurons and glia are gelatinases MMP-2 and MMP-9. MMP-9 was shown to affect long-term synaptic plasticity and memory consolidation [11, 13]. More recent data showed that stromelysin MMP-3 was upregulated in the hippocampus following learning and supported hippocampal synaptic plasticity [14–16]. In addition to modulating synaptic plasticity, a recent study found that acute and long-term extracellular proteolysis affected long-term neuronal excitability [10]. We recently found that the activity of certain MMP subtypes may modulate long-term NMDAR function, and MMP-3 and MMP-2/9 inhibition differentially affected the time course of E-S potentiation [17]. In the present study, we investigated the temporal relationships between the activity of these MMP subtypes, NMDAR function, and E-S potentiation in the CA1 region of the hippocampus. We found that the temporal requirement for NMDAR and MMP activity with regard to the expression of E-S potentiation largely overlapped and was still detectable approximately 15-30 min post tetanic stimulation. We also found that synaptic NMDAR function and postsynaptic Ca^2+^ entry were specifically regulated by MMP-3. Moreover, the magnitude of E-S potentiation following MMP-3 or NMDAR inhibition largely correlated with the expression of the nuclear protein cFos in CA1 pyramidal neurons, a marker of activity-related gene transcription. Altogether, we propose that MMP-3 activity may support long-term hippocampal E-S plasticity by promoting NMDAR-related postsynaptic Ca^2+^ entry and downstream intracellular cascades that are involved in activity-regulated gene expression. These results provide insights into the cellular mechanism of action of MMP on hippocampal plasticity that may open new lines of investigation on the rapid modulation of ionotropic synaptic NMDARs and Ca^2+^ entry by extracellular MMP.

## Materials and Methods

### Acute Brain Slice Electrophysiology

**Fig. 1:**
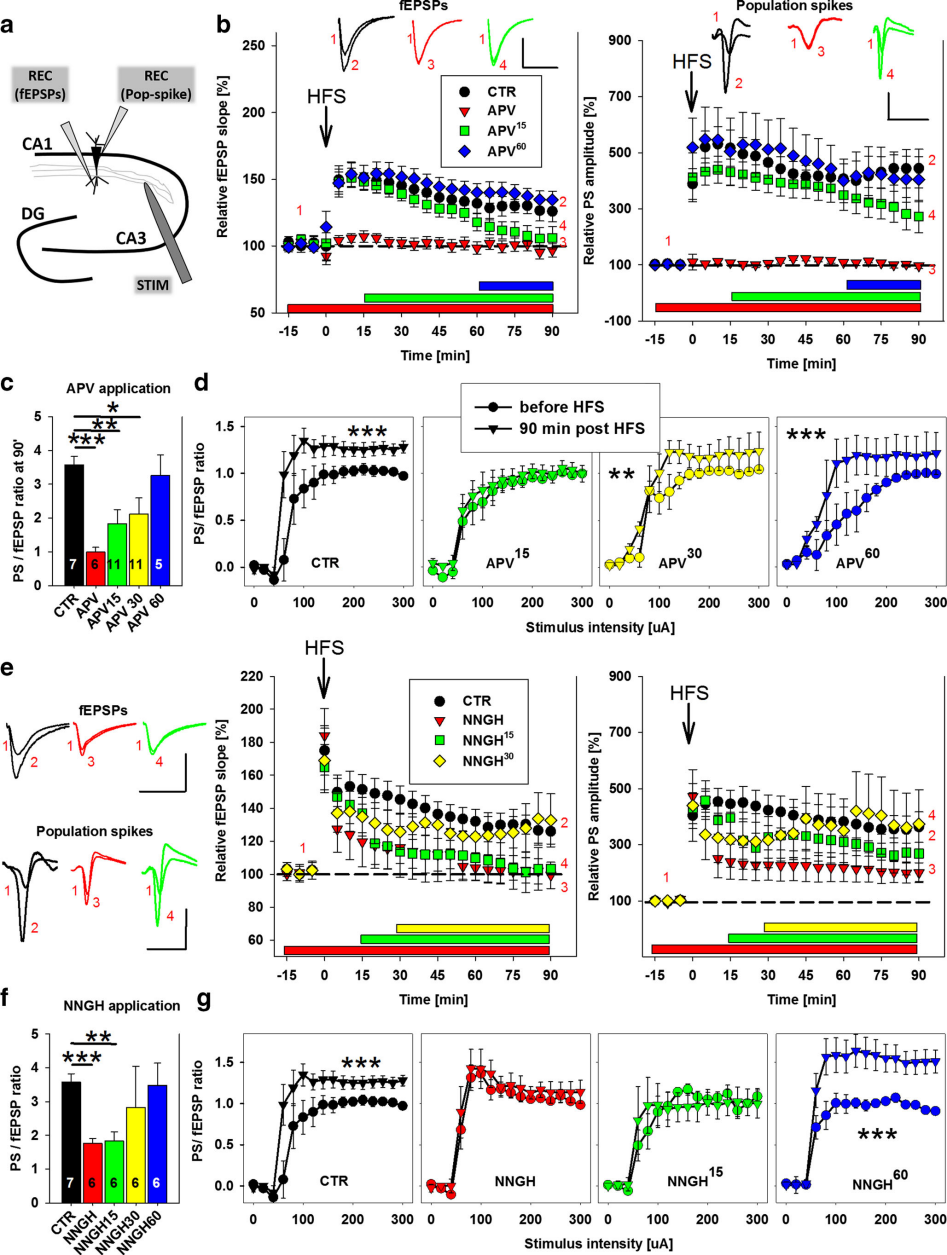
Temporal dependence of hippocampal E-S potentiation on NMDARs and MMP-3 activity. **a** Recording scheme. Two recording (REC) electrodes simultaneously monitored fEPSPs and population spikes in the CA1 region in response to Schaffer collateral stimulation (STIM). **b** *Left*, Time-course of maximal fEPSP slopes normalized to baseline values in control slices (*black circles*) and when bath-applied with the NMDAR antagonist APV (50 μM) at varying time points relative to HFS: before HFS (*red triangles*), 15 min post-HFS (*green squares*), or 60 min post-HFS (*blue diamonds*). The top shows exemplary traces of fEPSPs before HFS (*1*) and 90 min after HFS (colors match figure legend). *Scale bar* = 0.5 mV, 20 ms. *Right*, Time-course of population spike (PS) amplitudes normalized to baseline values in control slices (*black circles*) and when bath-applied with the NMDAR antagonist APV (50 μM) at varying time points relative to HFS: before HFS (*red triangles*), 15 min post-HFS (*green squares*), or 60 min post-HFS (*blue diamonds*). The top shows exemplary and normalized traces of PS before HFS (*1*) and 90 min after HFS (colors match figure legend). *Scale bar* = 0.5 mV, 10 ms. **c** Statistics of average PS/fEPSP ratio presented in **b** at 90 min post-HFS. The *asterisk* indicates a significant difference vs. slices in which HFS was applied in the absence of APV. Notice that APV that was applied up to 30 min post-HFS (*green*) significantly attenuated the PS/fEPSP upregulation following HFS. **d** Impact of APV on E-S coupling before HFS (*circles*) and 90 min after HFS (*triangles*). Relationships between stimulus strength and the PS/fEPSP slope ratio are shown. Notice that APV that was applied up to 30 min post-HFS (*yellow*) significantly attenuated the PS/fEPSP upregulation following HFS that was observed in CTR (*black*). **e** Effect of MMP-3 inhibitor NNGH (10 μM) on E-S potentiation. *Middle* and *right*, fEPSP slope and population spike amplitude time-course, respectively, as in **b**, except instead of APV, an MMP-3 inhibitor was applied at varying time points with regards to HFS. *Left*, Exemplary traces of fEPSPs (*top*) and population spikes (*bottom*) before and 90 min after HFS in the presence of NNGH (colors match figure legends). *Scale bars* as in **b. f** Statistics of average PS/fEPSP ratio presented in **e** at 90 min post-HFS. The *asterisk* indicates a significant difference vs. slices in which HFS was applied in the absence of NNGH. Notice that NNGH that was applied up to 15 min post-HFS (*green*) significantly attenuated the PS/fEPSP upregulation following HFS. **g** Impact of NNGH on E-S coupling before HFS (*circles*) and 90 min after HFS (*triangles*). Relationships between stimulus strength and the PS/fEPSP slope ratio are shown. Notice that NNGH that was applied up to 15 min post-HFS (*green*) significantly attenuated the PS/fEPSP upregulation following HFS that was observed in CTR (*black*). The zero value on the time bars represents the moment of tetanization (HFS, 4 × 100 Hz). The *horizontal colored bars* represent drug application. The *numbers* on the graphs refer to the number of experiments. *p < 0.05

The electrophysiological studies were conducted with C57BL/6 mice 30–60 days after birth. Acute brain slices were prepared as described previously [17]. All of the experimental procedures were approved by the Local Ethics Committee. Recordings were made in artificial cerebrospinal fluid (aCSF) that consisted of the following: 125 mM NaCl, 25 mM NaHCO_3_, 2.6 mM KCl, 1.25 mM NaH_2_PO_4_, 2.5 mM CaCl_2_, and 20 mM glucose, pH 7.4, at a temperature of 31 °C. Schaffer collateral (SCH) axons were stimulated with a concentric bipolar electrode (0.1 Hz, 0.25 ms). fEPSPs were recorded with glass micropipettes that were filled with aCSF (1-3 MΩ resistance) in the stratum radiatum of the CA1 region (150–200 μm from the stratum pyramidale). Population spikes were simultaneously recorded with another electrode that was placed in the stratum pyramidale below on the same axis (Fig. 1a). NMDAR-mediated signals were isolated with the AMPA/kainate receptor antagonist DNQX (20 μM) and l-type calcium channel blocker nifedipine (20 μM) in Mg^2+^-free solutions, as described previously [17]. At the end of each recording, the NMDAR antagonist APV (50 μM) was used to confirm the origin of the recorded fEPSP_NMDA_. We used the following MMP inhibitors: MMP-3 inhibitors NNGH (10 μM) and UK356618 (2 μM) and MMP-2/9 inhibitor SB3CT (10 μM). All of the drugs were obtained from Sigma-Aldrich (Poland), Tocris (UK), and Merck/Calbiochem (USA). Linear peptide GRGDSP was purchased from Proteogenix (France). The electrophysiology data were analyzed using pClamp10.3 software (Molecular Devices, USA) and AxoGraphX software (developed by John Clements) as described previously [17].

### Immunofluorescence in Hippocampal Sections

Immediately after the electrophysiological recordings, the hippocampal slices were fixed in 4 % paraformaldehyde for 1 day, washed in phosphate-buffered saline (PBS), and cut into 40-μm-thick sections on a vibratome (LeicaVT1000S) in gelatin blocks that were held at 4 °C. The sections were incubated overnight with primary antibodies (anti-c-Fos antibody, sc-7202, 1:200, Santa Cruz Biotechnology, Santa Cruz, CA, USA; anti-NeuN antibody, 1:1000, MAB377, Millipore, USA) and 3 % normal horse serum (NHS) at 4 °C. The slices were then washed in PBS with 0.3 % Triton X-100 and incubated with secondary antibodies conjugated with AlexaFLuor488 or AlexaFluor633 (ThermoFisher Scientific, Molecular Probes, USA). The sections were visualized with a FluoView1000 confocal microscope (Olympus, Poland) with a dry ×40/0.95NA objective. For a given slice, cFos + neurons were analyzed as described previously [18] in three non-overlapping three-dimensional pictures of the CA1 area downstream of the stimulation electrode.

### Calcium Imaging in Hippocampal Neuronal Cultures

Hippocampal neuronal cultures were prepared as described previously [19]. Neurons (cultured 14 days in vitro) were incubated for 30 min with Fura2 acetoxymethyl ester (2 μM, ThermoFisher Scientific, Molecular Probes, USA). The cells were then extensively washed with Ringer solution that consisted of the following: 137 mM NaCl, 5 mM KCl, 2 mM CaCl_2_, 1 mM MgCl_2_, 20 mM glucose, and 10 mM HEPES (pH 7.3). Recordings were made in Ringer solution in a microscope environmental chamber at 37 °C. Changes in intracellular calcium levels were measured using a Leica AF7000 Live Imaging System (Leica Microsystems GmbH, Wetzlar, Germany) with a ×20/0.7NA objective. Pairs of background-subtracted intensity images (16 bit, 502 × 501 pixels) were captured every 5 s at 510 nm (340 and 380 nm excitation wavelengths). To record Ca^2+^ waves that were associated solely with NMDARs, the recording solution was supplemented with the sodium channel blocker tetrodotoxin (1 μM), GABA_A_ receptor blocker picrotoxin (10 μM), AMPA/kainate receptor blocker DNQX (10 μM), l-type calcium channel blocker nifedipine (10 μM), and NMDAR co-agonists d-serine and glycine (1 μM). NMDA (60 μM) was applied for 60 s, and the sections were then extensively washed until the ΔF_340_/F_380_ signal returned to baseline (typically 5 min). To discriminate neurons from astrocytes, KCl (60 mM) was applied at the end of Fura2 imaging. Rapid shift in resting membrane potential toward depolarization and Ca^2+^ wave is observed in these conditions primarily in neurons rather than astrocytes [20]. Recombinant human MMP-3 (MBS142425, MyBioSource, Belgium) was activated according to the manufacturer’s instructions and tested for specific activity with a fluorogenic peptide substrate (ES002, R&D Systems, USA) and fluorescence plate reader (EnSpire Multimode Plate Reader, PerkinElmer, USA).

### Glutamate-Evoked Field Potential Recordings

**Fig. 2:**
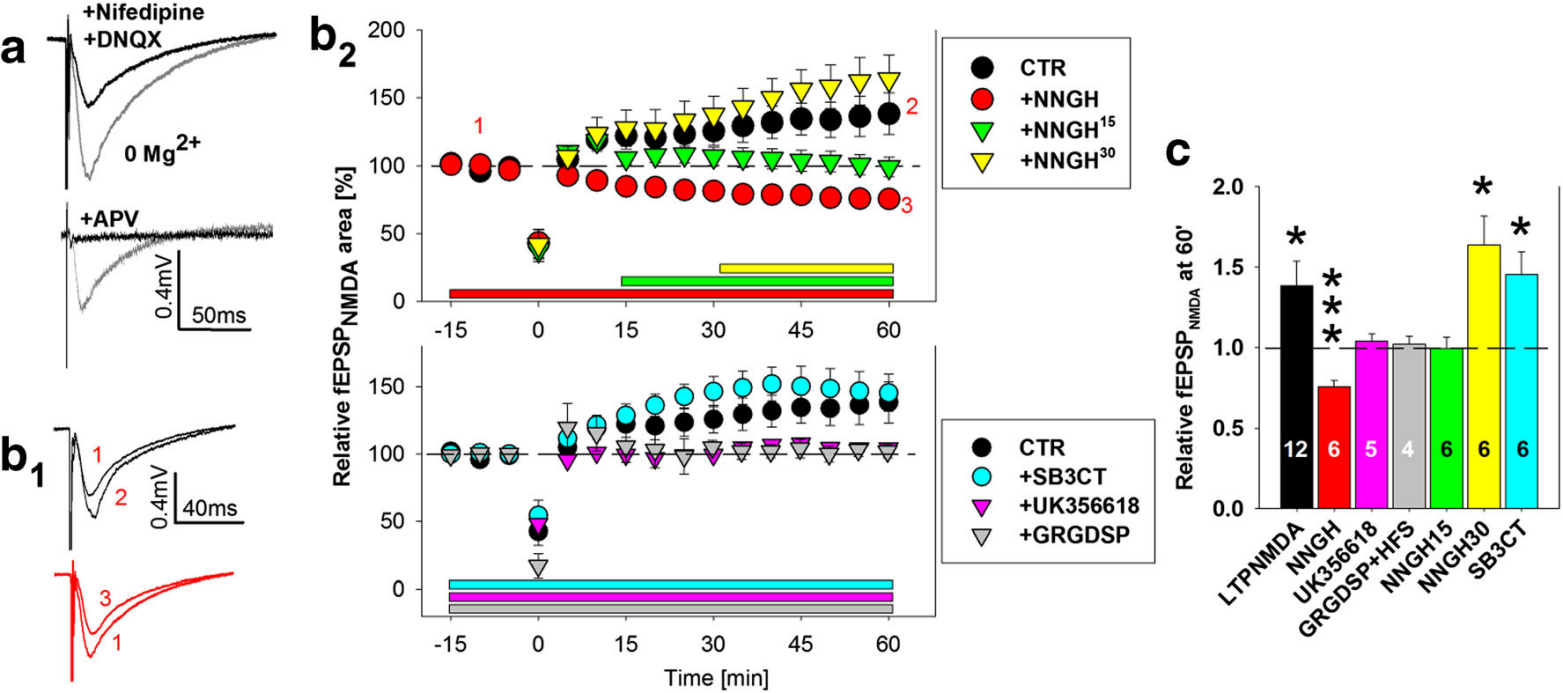
Synaptic NMDAR potentiation critically depends on MMP-3 and integrin signaling but not MMP-2/9. (*a*) Isolation of synaptic NMDAR-mediated fEPSPs (fEPSP_NMDA_). *Upper*, Sample traces of fEPSPs recorded in Mg^2+^-free solution before (*gray*) and after (*black*) application of the AMPA/kainate antagonist DNQX (20 μM) and l-type channel blocker nifedipine (20 μM). *Lower*, fEPSP_NMDA_ (*gray*) was completely abolished upon bath application of APV (50 μM) (*black*). (*b*_1_) Exemplary traces of fEPSP_NMDA_ before HFS (1) and 90 min after HFS (colors match (*b*_2_) legend). *Scale bar* = 0.2 mV, 20 ms. (*b*_2_) *Upper*, Time-course of synaptic NMDAR-mediated fEPSP under control conditions (*black*) and upon bath application of MMP-3 inhibitor before HFS (*red*), 15 min post-HFS (*green*), or 30 min post-HFS (*yellow*). *Lower*, Time-course of synaptic NMDAR-mediated fEPSP under control conditions (*black*) and upon bath application of drugs before HFS: MMP-3 inhibitor UK356618 (2 μM) (*magenta*), MMP-2/9 inhibitor (10 μM) (*cyan*), or integrin-blocking peptide GRGDSP (30 min post-HFS) (*gray*). The zero value on the *time bars* represents the moment of tetanization (HFS, 4 × 100 Hz). The *horizontal colored bars* represent drug application. (*c*) Statistics of effects of drugs shown in (*b*_2_) measured 60 min post-HFS. The *numbers* on the graphs refer to the number of experiments. *p < 0.05

Recordings of glutamate-evoked NMDAR-mediated field potentials were performed in acute brain slices in two steps. We first pharmacologically isolated NMDAR-mediated signals as described previously [17] (Fig. 2). After confirming the occurrence of synaptic NMDAR responses in the CA1 stratum radiatum, a field-potential recording pipette was approached with patch pipette (4-6 MΩ tip resistance) that was filled with glutamate (1 mM) and the synaptic NMDAR co-agonist d-serine (100 μM; Fig. 4a). Field potentials were recorded in response to short (200 ms) local application of the pipette solution via the Picospritzer®III system (Parker-Hannifin, USA; 6 PSI valve pressure).

Image analysis was performed using Fiji software [21]. The statistical analysis was performed using Student’s *t* test and analysis of variance (ANOVA), followed by post hoc tests or *χ* ^2^ tests as appropriate. The analyses were performed using SigmaStat 3.1 software (Systat Software). The data are expressed as mean ± SEM. The level of significance was *p* < 0.05.

## Results

### NMDAR Activity Supports E-S Plasticity Beyond an Episode of Enhanced Synaptic Activity

We first studied the temporal requirement for NMDAR activity in long-term E-S potentiation in the CA1 region of the hippocampus. We stimulated the SCH and recorded fEPSPs and population spikes (PSs) in the CA1 strata radiatum and pyramidale (Fig. 1a). Following basal stimulation (0.1 Hz, 15 min), we applied tetanic high-frequency stimulation (HFS; 4 × 100 Hz, 1 s, repeated every 10 s) and monitored the signals for the next 90 min. In control slices, HFS significantly potentiated synaptic fEPSPs and caused even more pronounced enhancement of the PS amplitude (*n* = 7 slices; *p* < 0.01 vs. baseline signals before HFS, Fig. 1b). Thus, the PS/fEPSP ratio, a measure of E-S potentiation, was 3.57 ± 0.24 (*n* = 7; *p* < 0.001 vs. baseline PS/fEPSP ratio, Fig. 1c).

We next bath-applied the competitive NMDAR antagonist APV (50 μM) at different time points relative to HFS. The application of APV 15 min before HFS completely abolished the potentiation of both fEPSPs and PSs and PS/fEPSP ratio was significantly reduced compared to CTR values (*n* = 6, *p* < 0.001, Fig. 1b, c). Surprisingly, the application of APV 15 or 30 min post-HFS (APV^15^ and APV^30^, respectively) destabilized fEPSP potentiation and reduced it to baseline levels, whereas E-S coupling was only partially attenuated (*n* = 11; *p* < 0.001 vs. CTR; Fig. 1b, c). Such an effect was not observed when APV was applied 60 min post-LTP induction (*n* = 5; *p* = 0.51 vs. CTR; Fig. 1c).

E-S coupling was then characterized over a wide range of monotonically increasing stimuli (0-300 μA) before and 90 min after HFS. As shown in Fig. 1d, significant upregulation of E-S coupling was observed in CTR slices (*F*_1,181_ = 33.04, *p* < 0.001), indicating enhanced postsynaptic spiking beyond that predicted solely by the increase in synaptic drive. In APV^15^ slices, we did not observe any statistically significant shift in the E-S curve (*F*_1,285_ = 2.33, *p* = 0.128). However, a longer gap between tetanization and APV administration resulted in a weaker effect of APV on the potentiation of E-S coupling, demonstrated by both APV^30^ and APV^60^ slices (*F*_1,285_ = 14.9 and *F*_1,129_ = 20, respectively; *p* = 0.01 and *p* = 0.001; Fig. 1d). Altogether, NMDAR activity was necessary for the expression of E-S potentiation within the time window of 15–30 min post patterned synaptic activity in the hippocampal CA1 region.

### MMP Activity Blockade Interferes with E-S Coupling Within a Temporal Window Similar to NMDAR Activity

We recently found that E-S potentiation within the hippocampal CA3 associational network critically depends on MMP activity, and MMP-3 may play a particularly important role in the time-course of this process [17]. However, the impact of MMP-3 inhibition on E-S coupling in the SCH-CA1 has not been previously studied. Therefore, we analyzed E-S potentiation in the presence of the bath-applied MMP-3 inhibitor NNGH (10 μM). Upon basal stimulation, neither fEPSPs nor PSs were affected by NNGH (data not shown). However, HFS-induced fEPSP slope potentiation was abolished (*n* = 6; *p* = 0.75 vs. baseline; Fig. 1e), whereas PS amplitude upregulation was reduced but not eliminated (*n* = 6; *p* = 0.01 vs. baseline; Fig. 1e). Altogether, the PS/fEPSP ratio that was measured in the presence of NNGH was significantly less than control conditions (*p* < 0.001; Fig. 1f). We then bath-applied NNGH at same time points relative to HFS as described above in studies of APV. NNGH application 15 min post-HFS (NNGH^15^) significantly attenuated the potentiation of E-S coupling (*n* = 6, *p* = 0.01; Fig. 1f), but this effect was not observed when NNGH was applied 30 or 60 min post-LTP induction (both *n* = 6; *p* = 0.65 and 0.87, respectively; Fig. 1f).

We then calculated the change in E-S coupling in response to a wide range of monotonically increasing stimuli (as described above; Fig. 1g) in NNGH and NNGH^15^ slices, but no significant shift in the E-S curve was observed (both *n* = 6; *F*_1,155_ = 0.27 and 0.377, respectively; *p* = 0.67 and 0.54, respectively). However, in NNGH^30^ and NNGH^60^ slices, a significant E-S curve shift was observed (both *n* = 6; *F*_1,155_ = 6.56 and 51.63, respectively; *p* = 0.01 and *p* < 0.001, respectively; Fig. 1g). Thus, the MMP-3 inhibitor NNGH impaired E-S potentiation in the SCH-CA1 projection when it was applied before and up to 15–30 min after HFS.

### MMP-3 but not MMP-2/9 Activity Is Crucial for the Potentiation of NMDAR-Mediated fEPSPs Following LTP

Considering that E-S potentiation requires nearly overlapping activity time windows for MMP-3 and NMDARs, we hypothesized that MMP supports the function of NMDARs within the first 30 min following HFS. To test this hypothesis, we pharmacologically isolated synaptic NMDAR-mediated responses (fEPSP_NMDA_; Fig. 2(a); see “Materials and Methods” section) and analyzed their sensitivity to MMP inhibition. Notably, from the point of NMDARs conductance for Ca^2+^, the signal integral (total area) rather than point amplitude better describes changes in NMDAR function. Therefore, instead of analyzing the fEPSP_NMDA_ point amplitude, we analyzed the fEPSP_NMDA_ area. We found that fEPSP_NMDA_ underwent slowly developing potentiation following HFS (134 ± 15 % of the baseline fEPSP_NMDA_ area 60 min post-HFS; LTP_NMDA_, *n* = 12, *p* = 0.02; Fig. 2(b)). When NNGH was present throughout the recordings, the LTD of NMDAR responses was observed following HFS (*n* = 6; *p* < 0.001 vs. baseline; Fig. 2(b)).

We then studied the temporal aspects of LTP_NMDA_ modulation by MMP-3 and found that NNGH interfered with LTP_NMDA_ when it was applied 15 min post-HFS (*n* = 6; *p* = 0.89 vs. baseline; Fig. 2(b)) but had no effect when it was applied 30 min post-HFS (163.6 ± 18 % of baseline at 60 min, Fig. 2(b); 167.7 ± 24 % of baseline at 90 min post-HFS, data not shown; *n* = 6; *p* = 0.02 vs. baseline).

We subsequently investigated the specificity and mechanism of MMP-related fEPSP_NMDA_ potentiation. In contrast to the actions of NNGH, application of the MMP-2/9 inhibitor SB3CT (10 μM) did not affect LTP_NMDA_ (*n* = 6; *p* = 0.021; Fig. 2(b, c)). MMP-9 supports the late phase of LTP_AMPA_ [22]. Therefore, we prolonged the recordings of fEPSP_NMDA_ up to 90 min in the presence of SB3CT, but no effect on NMDAR potentiation was observed (fEPSP_NMDA_ area potentiation was 153 ± 20 % of baseline; *n* = 6; *p* = 0.036; data not shown). Importantly, LTP_NMDA_ was not observed in the presence of another MMP-3 inhibitor, UK356618 (2 μM, *n* = 5, *p* = 0.65; Fig. 2(b, c)). In summary, the gain of function of NMDARs following HFS depended on MMP-3 activity (within a limited time window) rather than MMP-2/9 activity.

The MMP-mediated proteolysis of extracellular proteins may generate peptides that interfere with the integrin signaling system (reviewed in [11, 12]). To determine whether integrin signaling is involved in LTP_NMDA_ in our system, we incubated slices with the integrin-interfering peptide GRGDSP (0.5 mM) 15 min before HFS. As shown in Fig. 2(b), the prior activation of integrins with GRGDSP prevented the HFS-induced enhancement of fEPSP_NMDA_ (*n* = 4; *p* = 0.76 vs. baseline before HFS). Thus, integrin signaling in our system may be involved in HFS-induced LTP_NMDA_.

### MMP-3 or NMDAR Inhibition Affects c-Fos Expression Following HFS

**Fig. 3:**
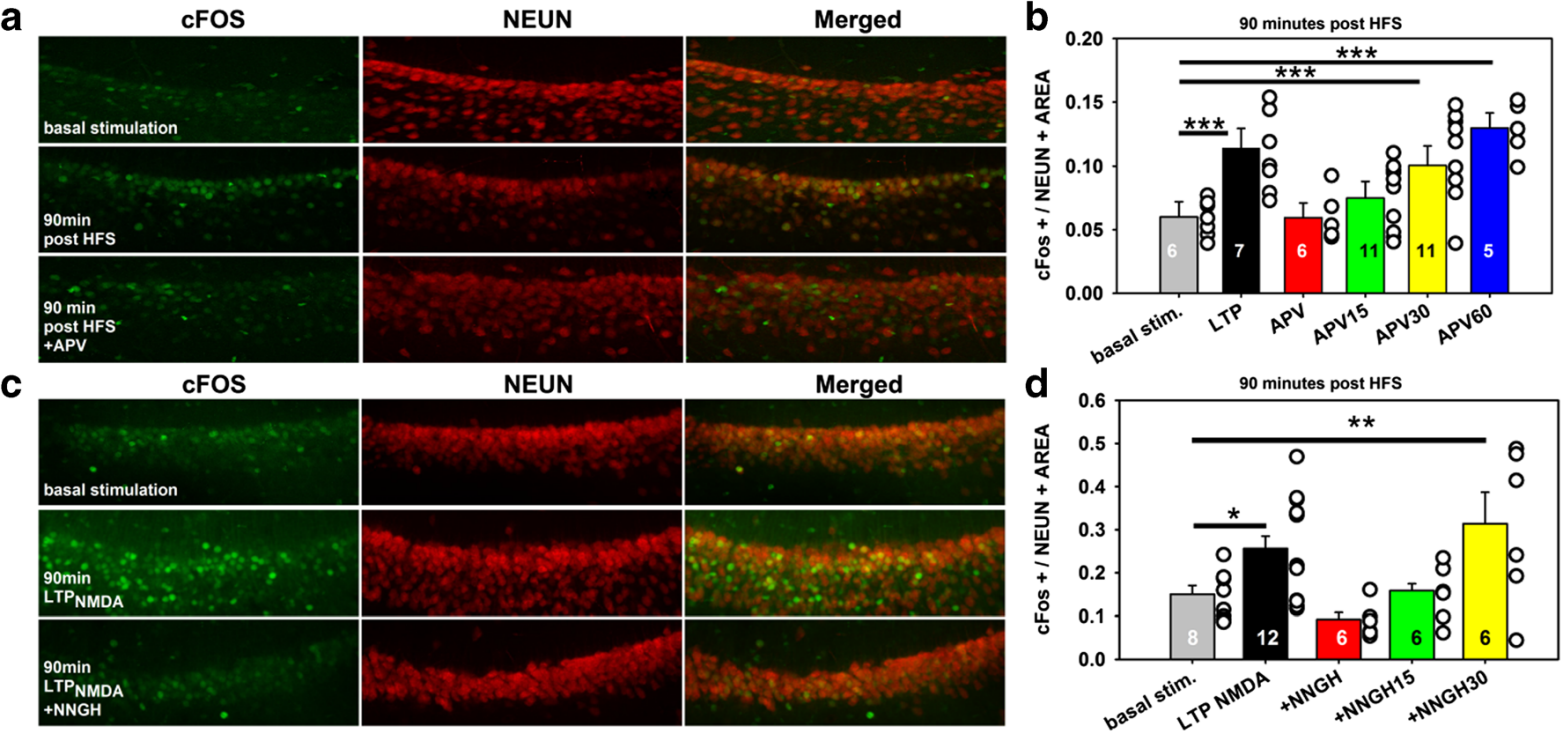
MMP-3 or NMDAR inhibition affects c-Fos expression following patterned neuronal activity. **a** Exemplary confocal immunofluorescence images of CA1 hippocampal region (×40 magnification) in acute brain slices fixed immediately following cessation of the electrophysiological recordings. Basally stimulated slices (0.1 Hz) (*upper panels*) were compared with slices fixed 90 min post-E-S potentiation induction in the absence (*middle panel*) or presence (*lower panel*) of APV. The sections were stained against cFos (*green channel*) and NeuN (*red channel*), and the colocalization of both markers was analyzed (*merged channel*). **b** Statistics of the effects of electrical stimulation and drug treatment on cFos expression in the CA1 neuronal population stained with NeuN. Slices were basally stimulated (0.1 Hz) (*gray*), or E-S potentiation was induced with HFS (4 × 100 Hz) in the absence (LTP) (*black*) or presence of the NMDAR antagonist APV applied before or 15 and 30 min after HFS. **c** Exemplary immunofluorescence images of CA1 sections following the induction of synaptic LTP_NMDA_ (see Fig. 2). Basally stimulated slices (0.1 Hz) (*upper panels*) were compared with slices that were fixed 90 min post-LTP_NMDA_ induction with HFS in the absence (*middle panel*) or presence (*lower panel*) of the MMP-3 inhibitor NNGH. See **a** for channel description. **d** Statistics of the effects of electrical stimulation and NNGH treatment on cFos expression in CA1 neurons following recordings of synaptic NMDAR-mediated responses (shown in Fig 2). Slices were basally stimulated (0.1 Hz) (*gray*), or LTP_NMDA_ was induced with HFS (4 × 100 Hz) in the absence (LTP) (*black*) or presence of the MMP-3 inhibitor NNGH applied before or 15 and 30 min after HFS. The *numbers* on the graphs refer to the number of sections analyzed. *p < 0.05 vs. basally stimulated slices

We hypothesized that impaired E-S potentiation and synaptic LTP_NMDA_ following MMP-3 inhibition may result in impaired activation of Ca^2+^-dependent intracellular cascades associated with the maintenance of long-term plasticity. Immediate early genes (IEGs), such as c-*fos*, and IEG-encoded transcription factors that affect target gene expression are often analyzed to map the activation of neurons in which the transcription of pro-plasticity genes is likely to be initiated [23, 24]. Therefore, we analyzed the expression of the nuclear protein cFos in CA1 neurons that were fixed immediately after cessation of the electrophysiological recordings that are shown in Figs. 1 and 2 (see “Materials and Methods” section). We evaluated slices in which the effects of APV on functional E-S plasticity were studied. As shown in Fig. 3a, b, under control conditions (i.e., basally stimulated slices and no HFS), only a small fraction of NeuN^+^ cells expressed cFos (*n* = 6). In contrast, in slices in which E-S potentiation was induced with HFS, a significant increase in the cFos^+^/NeuN^+^ fraction was observed compared with basally stimulated slices (*n* = 7; *p* < 0.001). In APV and APV^15^ slices, the cFos^+^/NeuN^+^ fraction was not significantly different from basally stimulated slices (*n* = 6 and 11, respectively; *p* = 0.947 and 0.218, respectively). However, in APV^30^ and APV^60^ slices, the cFos^+^/NeuN^+^ ratio was not significantly different from HFS-stimulated slices that were recorded in the absence of APV, unlike basally stimulated slices (*n* = 11 and 5, respectively; both *p* < 0.001; Fig. 3).

We next similarly analyzed slices in which LTP_NMDA_ was studied. As shown in Fig. 3c, d, under control conditions (i.e., basally stimulated slices and no HFS), the fraction of cFos^+^/NeuN^+^ neurons was 0.15 ± 0.02 (*n* = 8). In slices in which LTP_NMDA_ was induced, a significant increase in the cFos^+^/NeuN^+^ fraction was observed compared with basally stimulated slices (*n* = 12, *p* = 0.017). In NNGH and NNGH^15^ slices, the cFos^+^/NeuN^+^ was not significantly different from basally stimulated slices (both *n* = 6; *p* = 0.27 and 0.85, respectively vs. basally stimulated slices). In contrast, in NNGH^30^ slices, the cFos^+^/NeuN^+^ ratio was significantly potentiated compared only with basal stimulation (*n* = 6; *p* = 0.003). Thus, the magnitude of E-S potentiation and LTP_NMDA_ correlated with cFos expression in CA1 pyramidal neurons, and APV and NNGH influenced cFos expression when these drugs were applied before or up to 15 min post-HFS.

### Recombinant MMP-3 Promotes Glutamate-Evoked NMDAR Responses and Somatodendritic Ca^2+^ Waves Following Multiple Exposures to NMDA

**Fig. 4:**
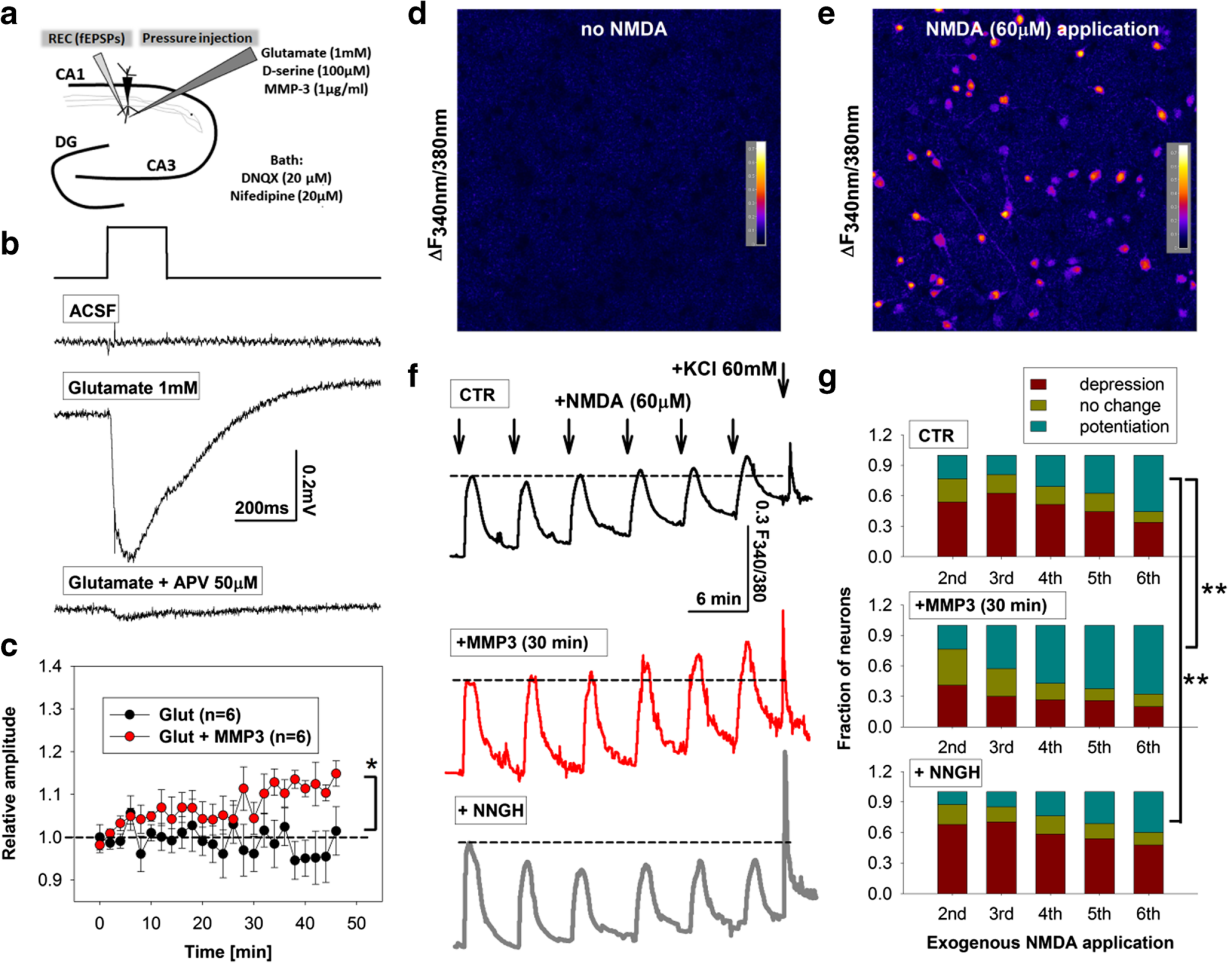
Recombinant MMP-3 promotes NMDAR-mediated responses and somatodendritic Ca^2+^ waves following multiple exposures to NMDA. **a** Recording scheme for glutamate-evoked NMDAR responses in acute hippocampal brain slice in the presence of inhibitor cocktail and Mg^2+^-free aCSF. A recording (REC) electrode recorded NMDAR-mediated field potentials in the CA1 stratum radiatum in response to local pressure injection of glutamate. **b** Representative NMDAR-mediated field potentials following short (200 ms) pressure injections of an external solution that contained the inhibitor cocktail (aCSF) (*upper trace*), aCSF + glutamate + d-serine (100 μM) without the NMDAR antagonist APV (*middle trace*), or aCSF + glutamate + d-serine with APV (*lower trace*). **c** Time-course of APV-sensitive field potential amplitude in response to glutamate (every 2 min) in the absence (*black circles*) or presence of recombinant MMP-3 (1 μg/ml). Notice the slowly emerging potentiation of field potential amplitude with time. **d**, **e** Exemplary differential images of relative Fura2 fluorescence change in somatodendritic compartment (*pink*) before (**d**) and during NMDA application (**e**, 60 μM; excitation wavelength, 340/380 nm; emission wavelength, 510 nm). The recording solution was supplemented with a cocktail of inhibitors to ensure NMDAR activation (see “Materials and Methods” section for details). **f** Exemplary time-course of Fura2 fluorescence (ΔF_340/380_) following exogenous application of NMDA (six applications every 5–6 min) in control neurons (*black trace*), neurons incubated with the MMP-3 inhibitor NNGH (10 μM) (*gray trace*), and neurons pretreated with recombinant MMP-3 (1 μg/ml) for 30 min before recordings (*red trace*). Notice that MMP-3 pretreatment typically enhanced the amplitude of NMDA-evoked Ca^2+^ waves, whereas MMP-3 inhibition reduced the amplitude of NMDA-evoked Ca^2+^ waves. **g** Statistics of data shown in **f** for all KCl-sensitive cells. The cells were classified as undergoing potentiation (>105 %) (*green*), no change (95 > 105 %) (*yellow*), or depression (<95 %) (*brown*) of NMDA-evoked Ca^2+^ wave amplitudes compared with first NMDA application. Notice that pretreatment with recombinant MMP-3 (*n* = 171 neurons, *n* = 3 cultures) (*middle panel*) increased the fraction of neurons that underwent Ca^2+^ wave amplitude potentiation with time (CTR, *n* = 408 neurons, *n* = 5 cultures; *χ* ^*2*^: *p* < 0.001, sixth vs. first) (*upper panel*). The MMP-3 inhibitor NNGH had the opposite effect (*n* = 395 neurons, *n* = 4 cultures) (*lower panel*). *p < 0.05 vs. control slices (sixth to first). *n* number of slices. *p < 0.05 vs. control slices

We next investigated the effect of recombinant MMP-3 on NMDAR function in acute brain slices. We recorded NMDAR-mediated local field potentials that were evoked by pressure-injected glutamate and d-serine in the presence of DNQX and nifedipine (see “Materials and Methods” section for details; Fig. 4a). Typical glutamate-evoked field potentials had an amplitude of 0.4 ± 0.1 mV (*n* = 6 slices) and were sensitive to APV application (Fig. 4b), indicating an NMDAR origin. Pressure application of the bath solution alone did not result in a detectable field potential (Fig. 4b, aCSF). Multiple applications of glutamate (every 2 min) for up to 45 min, together with recombinant MMP-3 (1 μg/ml), yielded a slowly emerging potentiation of NMDAR-mediated field potential amplitude (1.16 ± 0.03 relative change after 45 min of recording vs. baseline recorded in the first 6 min) compared with glutamate alone (0.96 ± 0.06, *n* = 6 slices, *p* = 0.026; Fig. 4c).

We next investigated whether MMP-3 activity affects NMDAR-mediated Ca^2+^ flux. We analyzed somatodendritic Ca^2+^ waves that were evoked by the exogenous application of NMDA in cultured hippocampal neurons with the ratiometric indicator Fura2 (see “Materials and Methods” section for details; Fig. 4d, e). As shown in Fig. 4f, NMDA application (60 μM, 60 s) significantly increased ΔF_340/380_ fluorescence in the somatodendritic compartment. The average amplitude of the first NMDA-evoked Ca^2+^ wave was Δ*F*_340/380_ = 0.30 ± 0.02 (*n* = 408 neurons, *n* = 5 cultures). Following multiple NMDA applications every 5–6 min, the neurons exhibited significant potentiation (>105 %, 55.5 % of all neurons; Fig. 4f, g), no change (95 > 105 %, 10 % of all neurons or depression (<95 %, 33.5 % of all neurons) of NMDA-evoked Ca^2+^ wave amplitudes compared with the first NMDA response (Fig. 4g). We then incubated the cultures with recombinant MMP-3 protein (1 μg/ml) for 30 min prior to Fura2 imaging (see “Materials and Methods” section for details) and grouped neurons based on the same criteria. This pretreatment did not significantly change the basal Ca^2+^ waves that were evoked by NMDA (first NMDA-mediated Ca^2+^ response, Δ*F*_340_/*F*_380_ = 0.29 ± 0.02, *n* = 171 neurons, *p* = 0.25). However, following multiple NMDA applications, a significant increase was observed in the fraction of neurons that exhibited potentiation of the Ca^2+^ wave amplitude (sixth vs. first application, *n* = 171 neurons, *n* = 3 cultures, *χ* ^2^ = 9.88, *p* = 0.007; Fig. 4g). We next incubated neuronal cultures with NNGH during exposures to NMDA. This treatment did not significantly change the first NMDA-mediated Ca^2+^ response (Δ*F*_340_/*F*_380_ = 0.29 ± 0.014, *n* = 395 neurons, *p* = 0.25). In contrast, in the presence of NNGH, significantly fewer neurons exhibited potentiation of the Ca^2+^ wave amplitude (sixth vs. first application, *n* = 395 neurons, *n* = 4 cultures, *χ* ^2^ = 11.86, *p* = 0.002; Fig. 4g). Altogether, exogenous MMP-3 enhanced somatodendritic Ca^2+^ waves following multiple exposures to NMDA in vitro and dendritic glutamate-evoked NMDAR-mediated field potentials in acute brain slices.

## Discussion

E-S potentiation occurs in vivo following certain learning paradigms and remains a cellular correlate of learning and memory (reviewed in [8, 25]). Although the exact cellular mechanism remains poorly understood, the activity of NMDARs appears to be particularly important in determining the magnitude of E-S potentiation [26, 27]. In the present study, we combined electrophysiology, immunocytochemistry, and Ca^2+^ imaging of hippocampal neurons to further investigate E-S plasticity’s temporal requirement for NMDARs and the extracellular activity of MMP-3 that was previously implicated as a potential modulator of NMDAR function. In the hippocampal CA1 field, long-term E-S potentiation appears to require transient MMP-3 activity that enhances NMDAR-mediated postsynaptic Ca^2+^ entry, which is vital for the activation of downstream signaling cascades and gene transcription.

### Prolonged NMDAR Activity Is Necessary for Maintaining E-S Potentiation

NMDARs fuel postsynaptic cells with Ca^2+^ mainly during episodes of enhanced neuronal activity and membrane depolarization. The present results suggest that long-term E-S potentiation following patterned neuronal stimulation in the hippocampal CA1 region requires temporally overlapping NMDAR and MMP activity. The magnitude of E-S potentiation depended on NMDAR activity for as long as ∼20–30 min post neuronal activity (Fig. 1), which is consistent with other experimental models. For example, the surface expression of GluN1 and GluN2A subunits increased and peaked 30 min post-HFS [28]. NMDAR activation was also shown to support synaptic LTP within the 30 min time window after induction in neurons in the developing visual system in *Xenopus laevis* [29]. In vivo, following learning in a passive avoidance task in chickens, an increase in NMDA binding to brain synaptosomal membranes was observed 30 min following passive avoidance training [30], and upregulation of the GluN1 and GluN2A NMDAR subunits was observed in reach training [31] and open field exploration [22].

The temporal requirement for NMDAR activity in E-S plasticity largely overlapped with the requirement for MMP-3 activity (Fig. 1). Additionally, we and others previously found that broad MMP inhibition or inhibition of MMP-9 in particular had no effect on synaptic LTP when performed approximately 30 min post-HFS [32–34]. If MMP-3 functions upstream of NMDAR in our system, then this would require the rapid release and sustained availability of MMP-3 for 15–30 min post-HFS. This is plausible because the immunoreactivity of MMP-9 and MMP-3 proteins and expression of MMP-9 and MMP-3 mRNA transcripts were previously observed in neuronal dendrites [35, 36]. Moreover, MMP-9 was shown to be rapidly (within a few minutes) and locally translated following neuronal activity [37].

### MMP-3 Activity Promotes NMDAR-Mediated Ca^2+^ Entry and cFos Expression

Based on the results presented in Figs. 2 and 4, we propose that MMP-3 may promote E-S plasticity by modulating NMDAR function and NMDAR-mediated Ca^2+^ influx, which may reveal a possible link between extracellular MMP activity and neuronal plasticity. Notably, both synaptic plasticity and the plasticity of endogenous excitability require a rise in Ca^2+^ [7]. With regard to neuronal excitability, NMDAR-mediated Ca^2+^ flow affects the activity of calcium-calmodulin kinase II (CaMKII) and protein synthesis that is crucial for the LTP of intrinsic excitability [38, 39]. NMDAR-mediated Ca^2+^ flux regulates hyperpolarization-activated cationic current (*I*_h_), which is crucial for dendritic excitability and the magnitude of E-S potentiation [26, 27]. Moreover, the extent of NMDAR-mediated membrane depolarization is expected to determine the rate of the subsequent activity of voltage-gated conductances and effectiveness of synaptic summation. Thus, the bursting activity of pyramidal cells is affected by at least two functionally opposite processes. While Ca^2+^ influx promotes membrane depolarization, it also enhances outward K^+^ currents via Ca^2+^-dependent potassium channels. The outcome of this current interplay determines the occurrence and duration of bursts in pyramidal neurons [40].

The long-term maintenance of memory traces requires a rise in Ca^2+^ and the transcription of pro-plasticity genes [41]. Therefore, we were interested in the extent to which NMDARs and MMP-3 activity may affect the level of activation of intracellular pathways that are critical for LTP in our system. We analyzed the expression of cFos protein, a product of the IEG c-*fos* (Fig. 3), because its induction was previously largely ascribed to NMDAR-mediated Ca^2+^ flux [42]. cFos expression was previously investigated to evaluate the activation of intracellular activity-triggered pathways and found to be important for experience-dependent neuronal development and plasticity [43, 44]. In the present study, the magnitude of E-S potentiation following the manipulation of NMDAR or MMP-3 activity correlated with cFos expression, suggesting a correlation with the level of activation of intracellular cascades that converge on gene transcription (Figs. 1 and 3). cFos induction was mainly triggered by NMDAR-mediated Ca^2+^ entry, demonstrated by the finding that we blocked l-type voltage-gated channel activity with nifedipine. Moreover, the washout of Mg^2+^ to promote NMDAR activation upregulated the basal proportion of neurons that expressed cFos following HFS (Fig. 3c, d). However, in addition to Ca^2+^ ions, several other molecules (e.g., brain-derived neurotrophic factor [BDNF]), have been implicated in triggering cFos expression (for review, see [23]). Additionally, E-S potentiation was affected by APV application for 30 min, but cFos expression was not (Figs. 2 and 3). This result can be explained by the fact that although NMDARs remain crucial for IEG expression, the latter may be additionally altered by the activity of non-NMDAR ionotropic and metabotropic receptors. Thus, we cannot exclude the possibility that HFS activated other pathways that are important for cFos expression. Finally, the AP1 transcription factor binding site is present in the promoter region of many MMP genes [45, 46], and the overexpression of cFos-containing AP-1 dimers induced MMP-9 transcription in neurons [47]. Thus, we speculate that the downregulation of MMP-3 activity might additionally suppress long-term E-S plasticity by negatively impacting the expression of pro-plasticity proteins and other MMPs. Matrix metalloproteases cleave proBDNF into mature BDNF, which can occur not only through the regulation of NMDAR Ca^2+^ flux but also through the proteolysis of extracellular factors [47, 48].

### MMP Subtype-Specific Modulation of E-S Plasticity and LTP_NMDA_

We recently reported that MMP-3 and MMP-2/9 activity remains crucial for E-S plasticity in the CA3 hippocampal circuit, but the effects of inhibiting these MMPs on the time-course of E-S LTP were clearly different [17]. The present results support the view that neuronal plasticity is expressed in neuronal compartments that are differentially sensitive to MMP. The MMP-3 inhibitor NNGH abolished CA1 synaptic LTP (Fig. 1, see also [14]) but the potentiation of PS amplitude was not completely abolished by this drug (Fig. 1e). This may suggest that MMP-3 functions mainly in the perisynaptic space rather than in the somatic space (as shown for MMP-9; [49]). Alternatively, because PS potentiation relies on excitation/inhibition balance [10], local changes in GABAergic inhibition following HFS may have more of an impact on PS plasticity than on fEPSP plasticity. This issue will require further investigation.

Several MMP subtypes were previously implicated in the interaction with NMDARs in vitro. Notably, MMP-7 but not MMP-1 or MMP-9 was shown to cleave the NR1 subunit of NMDARs and reduce NMDA-induced Ca^2+^ waves in acute brain slices [50]. Additionally, MMP-3 or MMP-7 cleaved NR1 and NR2A NMDAR subunits in vitro [50, 51]. MMP-9 also reversibly altered the kinetics of NMDAR-mediated currents [52] and promoted the lateral diffusion of these receptors in vitro [19]. The present study provides additional functional data by indicating that MMP-3 rather than MMP-2/9 activity may promote NMDAR function and NMDAR-mediated Ca^2+^ entry in vitro (Figs. 2 and 4). The latter finding corroborates a recent study in which broad MMP activity was implicated in supporting NMDA-stimulated Ca^2+^ waves in striatal dopaminergic medium spiny neurons [53]. MMP-3-mediated proteolysis may regulate the activity of other proteases, including MMP-9 and MMP-13 [54], emphasizing the importance of MMP-3 activity in NMDAR-dependent types of neural plasticity, learning, and memory [11, 12, 17].

Whether MMP-3 regulates NMDAR function directly or indirectly remains unknown. The ability of some MMP subtypes to cleave NMDAR subunits may suggest a direct interaction between MMPs and NMDARs [50, 51]. However, an equally likely possibility is that MMP-3 may indirectly upregulate NMDARs via protein kinase C and non-receptor tyrosine kinase Src activity that mediate LTP_NMDA_ [28]. This could be achieved by activating such transmembrane proteins as integrins and intracellular adhesion molecule-5 and also protease-activated receptor 1, which were previously shown downstream MMP activity and to affect Src and PKC activity [14, 32, 55, 56]. The involvement of integrin signaling is particularly likely because the MMP-3 cleavage of laminin/fibronectin [54] may provide integrin-activating peptides that contain the RGD motif. The integrin-blocking peptide GRGDSP was utilized to compete with the recognition site for a subclass of integrins that act as fibrinogen receptors, and GRGDSP prevented HFS-induced LTP_NMDA_ in our system (Fig. 2(b)). Additionally, integrin interaction with synthetic RGD peptides was shown to induce LTP_NMDA_ [56] and rapid Ca^2+^ waves [57] and interfere with synaptic LTP_AMPA_ up to 15 min post-LTP induction [58], similar to the effects of NNGH on E-S potentiation in the present study (Fig. 1). Future studies of isolated synaptic components (e.g., the NMDA component) may help narrow the list of potential signaling cascades that are activated by MMP.

Several aspects of the MMP/NMDAR interaction need further investigation. We used commercial MMP subtype-specific inhibitors. However, as discussed previously [11, 17], these drugs at high doses may act as broad-spectrum inhibitors. In the present study, NNGH (10 μM) application resulted in fEPSP_NMDA_ depression following HFS, and UK356618 (2 μM) resulted in a less pronounced effect, suggesting dose-dependent effects of these drugs or differences in MMP subtype specificity (Fig. 2). Thus, although we used two different MMP-3 inhibitors and recombinant MMP-3, we cannot exclude the possibility that other MMPs with similar substrate specificity to MMP-3 may play equally important roles in E-S potentiation. Another important issue is whether the MMP-3/NMDAR interaction is subunit-specific. The NR2A and NR2B subunits of NMDARs conduct significantly different amounts of Ca^2+^ [59]. Moreover, different pools of NMDARs are capable of causing distinct changes in gene transcription [60]. NMDAR activity has also been shown to be crucial in long-term associative memory in several learning paradigms [61]. Rapid hippocampal place fields formation [61] and the stability of CA1 spatial maps require the temporally matched activity of NMDARs [62]. Thus, the effects of MMP on NMDARs following patterned neuronal activity in the hippocampal CA1 region may alter the formation of place fields, which could explain the impairment in hippocampal spatial learning following MMP inhibition [63]. Additionally, NMDARs and MMP activity have been implicated in several neurological and psychiatric disorders, such as ischemia, epilepsy, schizophrenia, drug addiction, and neurodegenerative diseases [3, 64]. Particularly important, MMP-3 activity has been implicated in the pathophysiology of Parkinson’s disease, Alzheimer’s disease, and ischemic neuronal death [65]. Therefore, one possibility is that the coupling of MMP-3 and NMDAR activity that was found in the present study may operate beyond E-S plasticity.
